# One-to-one encapsulation based on alternating droplet generation

**DOI:** 10.1038/srep15196

**Published:** 2015-10-21

**Authors:** Hirotada Hirama, Toru Torii

**Affiliations:** 1 Department of Human and Engineered Environmental Studies, Graduate School of Frontier Sciences, The University of Tokyo, Kashiwa-shi, Chiba 277-8563, Japan

## Abstract

This paper reports the preparation of encapsulated particles as models of cells using an alternating droplet generation encapsulation method in which the number of particles in a droplet is controlled by a microchannel to achieve one-to-one encapsulation. Using a microchannel in which wettability is treated locally, the fluorescent particles used as models of cells were successfully encapsulated in uniform water-in-oil-in-water (W/O/W) emulsion droplets. Furthermore, 20% of the particle-containing droplets contained one particle. Additionally, when a surfactant with the appropriate properties was used, the fluorescent particles within each inner aqueous droplet were enclosed in the merged droplet by spontaneous droplet coalescence. This one-to-one encapsulation method based on alternating droplet generation could be used for a variety of applications, such as high-throughput single-cell assays, gene transfection into cells or one-to-one cell fusion.

Manipulation using microfluidics, a technology that enables handling small amounts of fluid (nano to atto litres)^1^, and single-cell sorting are promising technologies for high-throughput single-cell assays^2^,^3^,^4^,^5^, gene transfection into cells^6^, and one-to-one cell fusion^7^,^8^. In droplet microfluidics, the fluid is manipulated as a droplet, a small capsule that can be handled independently of the external environment by confining the reagents and/or particulate matter such as a functional particle, cell, or molecule. Droplets can be formed using a microchannel, which enables the continuous formation of massive numbers of droplets (e.g., ~10^3^ Hz), and the droplet size as well as the number and volume of materials confined in a droplet can be precisely controlled in a microchannel. Such droplets have been used as miniature compartments for chemical, physical, and biological reactions and have been implemented in high-throughput screening applications such as single-cell analysis^9^, combinatorial synthesis^10^, protein crystallisation condition screening^11^, and molecular evolution experiments^12^. To realise these applications, a variety of elemental technologies have been developed for the encapsulation of cells and particles^5^,^13^,^14^. However, in spite of these recent developments, a “high-throughput one-to-one cell encapsulation method” has not yet been realized, even though it could enable precise and rapid handling of one cell at a time.

Alternating droplet generation is one type of droplet generation technique that can be implemented using a microchannel^15^,^16^. This technique involves the combination of fluid vibration (“push-pull” mechanism) at the cross-junction of a microchannel^17^ and other factors (e.g., shear and interfacial tension). Specifically, using a microchannel with a cross-junction and a sheath-flow junction positioned adjacent to one another, multiple emulsion droplets can be formed. The smaller droplets that are generated at the cross-junction are enclosed within the larger droplets formed at the sheath-flow junction. This technique can be used to produce a variety of multiple emulsion droplets such as water-in-oil-in-water (W/O/W) emulsion droplets and oil-in-water-in-oil (O/W/O) emulsion droplets. These multiple emulsion droplets can be used to encapsulate various materials, including micro- and nanoparticles, biomaterials (e.g., proteins and nucleic acids), and cells.

Our group previously reported the use of a precise microfluidic control technique to achieve (1) confinement of particulate matter (i.e., a bead or a cell) in a droplet and (2) confinement of two different liquids in a multiple emulsion droplet. Combining these two techniques could enable the confinement of two different particles in a multiple emulsion droplet, i.e., one-to-one encapsulation, but this technique has not yet been demonstrated.

This paper reports a novel one-to-one encapsulation method with alternating droplet generation and droplet combination. The droplet generation was controlled to produce monodisperse multiple emulsion droplets encapsulating the desired number of controlled particles (as a model of cells) in a microchannel with partially controlled wettability. We also investigated the effects of surfactant addition to the organic phase to stabilise droplet formation and drive droplet combination to confine two different particles in a single droplet. A surfactant with the appropriate properties could be used to achieve one-to-one encapsulation in a multiple emulsion droplet, but this has not yet been reported. Therefore, we selected an appropriate surfactant by investigating various surfactants with a range of characteristics.

## Results

### Generation of W/O/W emulsion droplets using a wettability-controlled microchannel

**Figure 1:**
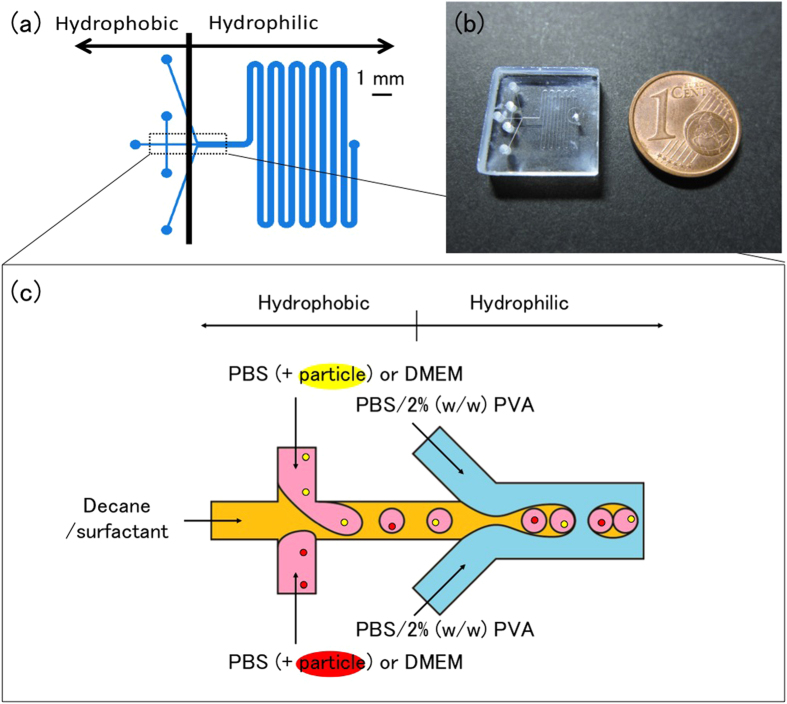
Microchannel used for W/O/W emulsion droplet formation. (**a**) Configuration and patterned wettability. (**b**) Fused silica glass microchannel chip. (**c**) Encapsulation process based on alternating droplet generation.

**Figure 2:**
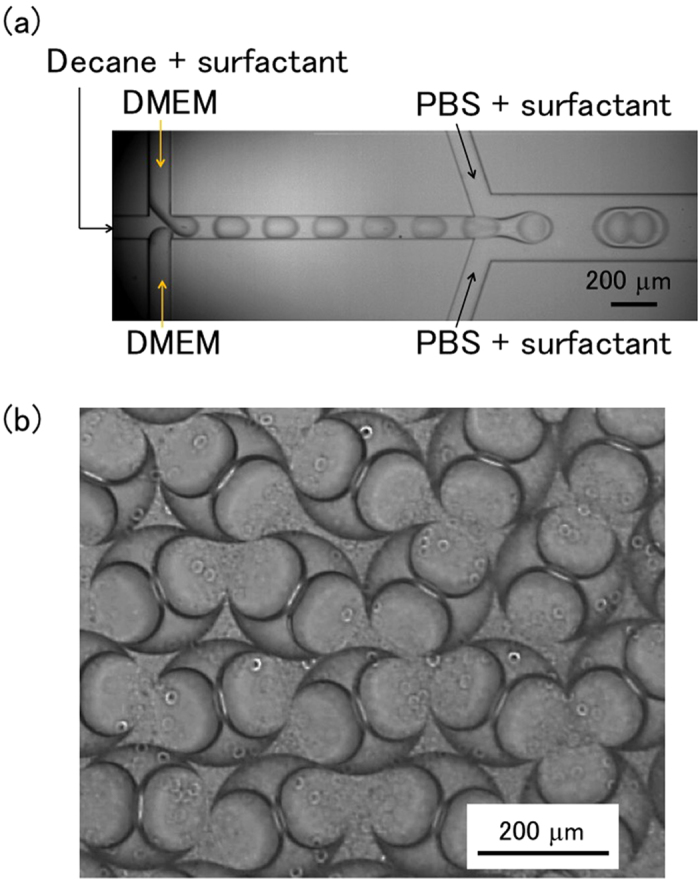
W/O/W emulsion droplet generation. (**a**) Droplet generation in the microchannel. (**b**) Generated droplets.

The aim of this study was to achieve one-to-one encapsulation using a W/O/W emulsion droplet, in which an aqueous droplet is contained within an organic droplet. As aqueous and organic droplets can be generated in hydrophobic and hydrophilic microchannels, respectively (Fig. 1), we fabricated a microchannel with a change in wettability (i.e., hydrophobic or hydrophilic) between the upstream and downstream portions of the channel by a partial wettability control method^16^,^18^ with silane, octadecyl trichlorosilane (OTS), and ultraviolet (UV) irradiation. Figure 2 and Supplementary Video S1 show W/O/W emulsion droplet formation with hydrophilic and hydrophobic microchannels. Droplets were formed by the following two steps: (1) in the upstream portion of the microchannel, inner aqueous (Dulbecco’s Modified Eagle’s Medium (DMEM)) droplets were formed in an organic (decane) droplet supplemented with surfactant, and (2) in the downstream portion of the microchannel, organic (decane) droplets containing the inner aqueous droplets were formed in phosphate buffered saline (PBS) supplemented with polyvinyl alcohol (PVA) surfactant. In this droplet formation occurring in the upstream portion of the microchannel, alternating droplet generation was observed in which the inner aqueous phase was constantly in motion in a “push-pull” mechanism^17^, resulting in one-by-one alternating droplet generation (Fig. 2a).

### Control of the number of inner aqueous droplets within a W/O/W emulsion droplet by adjusting flow rates towards one-to-one encapsulation

**Figure 3:**
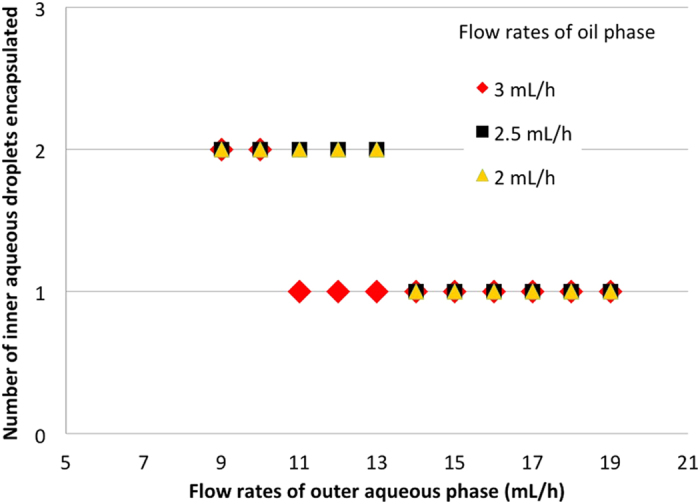
Relationship between the flow rate of the outer aqueous phase and the number of inner aqueous droplets that were encapsulated.

The number of inner aqueous droplets encapsulated in a W/O/W emulsion droplet can be controlled by flow rates of each solution introduced into the microchannel^16^. Here, we investigated the flow rates at which W/O/W emulsion droplets could be produced consisting of an organic droplet containing two inner aqueous droplets. At an inner aqueous phase flow rate of 1.5 mL/h and organic phase flow rates of 2, 2.5, or 3 mL/h, between one and two inner aqueous droplets were encapsulated in the organic droplets (Fig. 3), and droplets formed stably and continuously at outer aqueous phase flow rates ranging from 8–20 mL/h. In contrast, droplets could not be stably formed at outer aqueous phase flow rates of less than 8 mL/h or more than 20 mL/h, resulting in the formation of a range of droplet sizes. These results confirm the findings of a previous report^15^ that adjustment of the flow rates of the inner aqueous phase, organic phase, and outer aqueous phase enables the generation of a W/O/W emulsion droplet, an organic droplet containing two aqueous droplets.

### Relationship between surfactant type and the stability of W/O/W emulsion droplets

To achieve one-to-one encapsulation by generating a W/O/W emulsion droplet that contains two different materials (Fig. 1c), W/O/W emulsion droplets collected after generation in a microchannel had to meet the following conditions: (1) while organic droplets do not coalesce, inner aqueous droplets coalesce, and (2) inner aqueous droplets in an organic droplet are retained. As immediate droplet coalescence depends on the type of surfactant, we confirmed the relationship between the state of the W/O/W emulsion droplet that was collected and the type of surfactant that was added to the organic phase. Surfactant added to the organic phase was chosen based on the hydrophile-lipophile balance (HLB) value and whether the surfactant molecule was polymeric or monomeric (Table 1). The HLB value proposed by Griffin represents the hydrophilicity/hydrophobicity of the surfactant^19^. Generally, hydrophilic surfactants exhibit high HLB values and lipophilic surfactants exhibit low HLB values.

When the organic phase was supplemented with surfactant, stable W/O/W emulsion droplets were formed (Table 2b–e), while no W/O/W emulsion droplets were formed without the surfactant (Table 2a). When a surfactant with a relatively low HLB value (=1.8) was added to the organic phase, the W/O/W emulsion droplets broke off the microchannel shortly after collection (in a polystyrene petri dish) due to the release of the inner aqueous droplets to the outer aqueous phase (Table 2b). In contrast, when a surfactant with a relatively high HLB value (=3 or 4.3) was added to the organic phase, the resulting W/O/W emulsion droplets remained stable for more than several tens of minutes (Table 2c,d). When a mixture of both monomeric and polymeric surfactants was used, the W/O/W emulsion droplets broke shortly after collection in a polystyrene petri dish, leaving only organic droplets (Table 2e).

### Particle encapsulation towards one-to-one encapsulation

**Figure 4:**
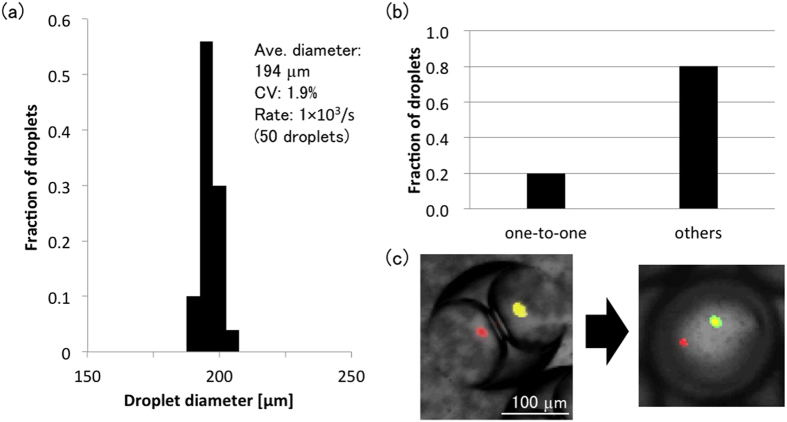
W/O/W emulsion droplets containing one fluorescent particle. (**a**) Histogram of the diameters of W/O/W emulsion droplets. (**b**) Fraction of droplets containing one particle (n = 106). (**c**) Confocal image of a one-to-one particle containing droplet. The image depicts before and after the coalescence of the inner aqueous droplets.

To form W/O/W emulsion droplets containing one-to-one fluorescent particles in inner aqueous droplets (as shown in Fig. 1c), we investigated W/O/W emulsion droplet formation where the inner aqueous phase (PBS) contained suspended fluorescent particles. Introducing the inner aqueous phase (flow rate: 1.5 mL/h), organic phase (flow rate: 3 mL/h), and outer aqueous phase (flow rate: 10 mL/h) yielded W/O/W emulsion droplets containing two aqueous droplets. The W/O/W emulsion droplets exhibited a mean diameter of 194 μm (standard deviation: 3.6 μm), a coefficient of variation (CV) of 1.9%, and a throughput of 1 × 10^3^ Hz (Fig. 4a). Twenty percent of the W/O/W emulsion droplets containing particles contained one-to-one particles (Fig. 4b). In addition, under these experimental conditions, the two inner aqueous droplets spontaneously merged after droplet generation, with the result that the fluorescent particles within each inner aqueous droplet were enclosed in the merged droplet (Fig. 4c). These results demonstrate that this encapsulation technique could be used as a high-throughput one-to-one cell encapsulation method.

## Discussion

As in a previous report^15^, W/O/W emulsion droplets were continuously formed in a microchannel using an organic phase supplemented with surfactant. According to the literature^20^, this phenomenon is due to the following surfactant behaviour. (1) Molecules of surfactant added to the continuous phase (i.e., the organic phase in this study) quickly adsorbed at the interface between the continuous phase and the dispersed phase (i.e., the inner aqueous phase in this study), resulting in the formation of a molecular film of the surfactant at the interface. (2) This surfactant film increased the mechanical strength of the droplet interface, resulting in the formation of stable droplets enclosed by the film. In an experiment where surfactant was not added to the organic phase, W/O/W emulsion droplets were not generated because no surfactant film was formed.

Differences in the stability of droplets ejected from a microchannel to a petri dish were assumed to result from differences in the surfactant HLB value, whether the surfactant was monomeric or polymeric, and the presence or absence of interactions among surfactant molecules at the droplet surface. First, the addition of surfactants with higher HLB values to the continuous phase (the organic phase in this experiment) was less likely to lead to droplet coalescence^20^. The experimental surfactant selection results for stable W/O/W emulsion droplet generation demonstrate that the boundary of the HLB value at which a droplet can be held without coalescence of organic droplets ranged from 1.8 to 4.3. Second, when either polymeric or monomeric surfactants are used, a film is formed at the droplet interface, making W/O/W emulsion droplets stable. In contrast, when a mixed surfactant is used containing both monomeric and polymeric surfactants, the surfactant film is destabilised^20^. Therefore, when both monomeric and polymeric surfactants were used, W/O/W emulsion droplets were not stably maintained. These results demonstrate that W/O/W emulsion droplets could be stably produced using a single surfactant (of either polymer or monomer) with an HLB value of 3 or 4.3. Under these conditions, the organic droplets did not coalesce and the inner aqueous droplets in the organic droplet were retained.

A previous report predicted the morphology of a liquid three-phase system (e.g., W/O/W emulsion droplet) at equilibrium using the spreading coefficient obtained by interfacial tension^21^,^22^. The spreading coefficient of the liquid, S, is expressed by the following equation using the interfacial tension, γ:


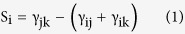


Using the following expansion Fowkes formula^23^, γ is determined from the surface tension, σ:


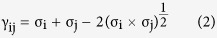


**Figure 5:**
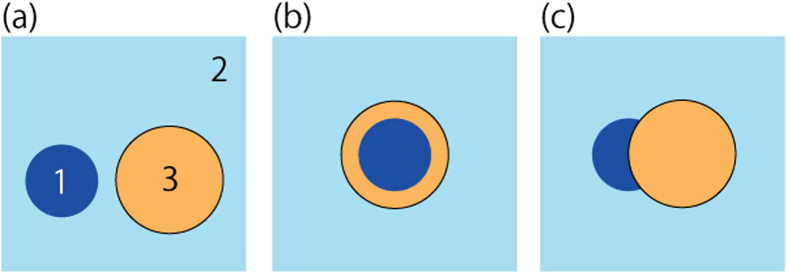
Schematic drawing of the morphology of the three-phase system used in this study. (**a**) Non-engulfing. Two droplets are independent. (**b**) Complete engulfing. A droplet completely contains another droplet. (**c**) Partial engulfing. A droplet partially contains another droplet.

From the positive and negative spreading coefficients obtained by measuring interfacial tension, the morphology of the three-phase system at equilibrium can be predicted as follows^21^ (note: the “continuous phase” was the outer aqueous phase in this experiment): (1) when S_1_ < 0, S_2_ > 0, S_3_ < 0, a droplet from phase 1 and a droplet from phase 3 exist independently in the continuous phase of phase 2 (non-engulfing) (Fig. 5a); (2) when S_1_ < 0, S_2_ < 0, S_3_ > 0, a droplet from phase 3 completely containing a droplet from phase 1 exists in the continuous phase of phase 2 (complete engulfing) (Figs 5b and 3) when S_1_ < 0, S_2_ < 0, S_3_ < 0, a droplet from phase 3 partially containing a droplet from phase 1 exists in the continuous phase of phase 2 (partial engulfing) (Fig. 5c). In this study, the surface tension was measured to evaluate the stability of the generated W/O/W emulsion droplets at equilibrium, and the positive and negative spreading coefficients were determined. Table 3 shows the surface tension of the liquid reagent that was used to form W/O/W emulsion droplets and determine the obtained spreading coefficients. For any combination of reagents in this study, a complete engulfing morphology could be formed at equilibrium. This result demonstrated that for the combination of reagents used in this study, the generated W/O/W emulsion droplets enabled the formation of a complete engulfing morphology in the equilibrium state. In contrast, the W/O/W emulsion droplets generated by adding SPAN 85 or both SPAN 80 and SY-Glyster CRS-75 to the organic phase formed a complete engulfing morphology according to the positive and negative spreading coefficients; however, these W/O/W emulsion droplets released the inner aqueous droplets, and the W/O/W emulsion droplets formed non-engulfing morphologies shortly after collection (as shown in Table 2b,e). This is because the effect of the surfactant molecules’ behaviour at the droplet interface became dominant compared with the effect of the spreading coefficient. Thus, when a single polymeric or monomeric surfactant with an HLB value of 3 or 4.3 was used, it was possible to produce stable W/O/W emulsion droplets containing two inner aqueous droplets.

In conclusion, we achieved one-to-one encapsulation by monodisperse formation of multiple droplets in a microchannel with partially controlled wettability, and by the coalescence of inner aqueous droplets driven by the surfactant properties (i.e., the HLB value and whether the surfactant was polymeric or monomeric). This method enables us to perform precise microencapsulation. By sorting one-to-one cells with a cell sorter after the encapsulation process, W/O/W emulsion droplets containing single cells could be utilized for various applications such as high-throughput single-cell assays, gene transfection into cells, and one-to-one cell fusion.

## Methods

### Surface modification to control the wettability of microchannels

To generate W/O/W emulsion droplets, the upstream and downstream portions of a microchannel (produced by deep reactive ion etching) were treated as hydrophobic and hydrophilic, respectively (as shown in Fig. 1a). This study used the partial wettability control method with a silane solution, toluene/1% (v/v) OTS, and UV irradiation^16^,^18^. To summarise the method, the entire microchannel is first made hydrophobic by filling the microchannel with OTS. Subsequently, to make only the downstream portion of the microchannel hydrophilic, only the downstream portion is irradiated with UV for 2 hours, with the upstream portion masked with aluminium. As a result, only the downstream portion of the microchannel is UV irradiated, forming silanol groups (Si-OH) and thus becoming hydrophobic.

### Droplet formation

The microchannel was set up for droplet formation as follows. All microchannels had a depth of 100 μm, with injection and drain channel widths of 100 μm and 300 μm, respectively. Each microchannel inlet/outlet was connected to a syringe (10 mL gastight syringe, Hamilton Company, USA) via polytetrafluoroethylene (PTFE) tubing (1.59 mm/0.5 mm outer/inner diameter, Flon Industry, Japan). To form droplets, each solution was filled into the syringes and introduced into each microchannel at constant flow rates using a syringe pump (KDS200, KD Scientific, USA). Droplet formation was observed using a high speed camera (FASTCAM-MAX 120K, Photron, Japan) mounted on an upright microscope (BX-50, Olympus, Japan).

### Selection of surfactant to enhance the stability of W/O/W emulsion droplets

We investigated the relationship between the HLB value of the surfactant added to the organic phase and the stability of the W/O/W emulsion droplets. The surfactants were SPAN 85 (sorbitan trioleate, HLB value: 1.8, monomer, Wako Pure Chemical Industries, Japan), SPAN 80 (sorbitan monooleate, HLB value: 4.3, monomer, Wako Pure Chemical Industries, Japan), and SY-Glyster CRS-75 (polyglycerol esters of fatty acids, HLB value: 3, polymer, Sakamoto Yakuhin Kogyo, Osaka, Japan). These three surfactants were used to prepare three different organic phases consisting of decane (Wako Pure Chemical Industries, Ltd., Japan)/1% surfactant. Additionally, to verify the status of droplet formation using a decane organic phase supplemented with both monomeric and polymeric surfactants, decane was supplemented with both 1% (w/w) SPAN 80 and 0.5% (w/w) SY-Glyster CRS-75. These organic phases, inner aqueous phases (DMEM, Life Technologies, USA), and outer aqueous phases (PBS/2% PVA (The Nippon Synthetic Chemical Industry Co., Ltd., Japan)) were filled into syringes and then introduced into the microchannel using syringe pumps. Droplet formation was observed using a high speed camera mounted on an upright microscope.

### Generation of W/O/W emulsion droplets containing fluorescent particles

To generate W/O/W emulsion droplets containing two types of fluorescent particles (made from polystyrene) with different excitation wavelengths (Fluoresbrite Polychromatic Red 6.0 Micron Microspheres and Fluoresbrite Yellow Green Microspheres 10 μm, Polysciences, Inc., USA), organic phases, inner aqueous phases, and outer aqueous phases were prepared and filled into syringes and then introduced into the microchannel using syringe pumps. The organic phase was decane/1% (w/w) SY-Glyster CRS-75, the inner aqueous phase was PBS containing fluorescent particles (10^5^ particles/mL), and the outer aqueous phase was PBS/2% (w/w) PVA. Droplet formation was observed using a high speed camera mounted on an upright microscope. W/O/W emulsion droplets containing fluorescent particles were imaged using a charge coupled device (CCD) camera mounted on an inverted microscope (IX-71, Olympus, Japan) and a confocal microscope system (FV-300, Olympus, Japan). Droplet sizes were measured using image analysis software (Image-Pro Plus, Media Cybernetics, USA).

## Additional Information

**How to cite this article**: Hirama, H. and Torii, T. One-to-one encapsulation based on alternating droplet generation. *Sci. Rep.* **5**, 15196; doi: 10.1038/srep15196 (2015).

## Supplementary Material

Supplementary Video S1

## Figures and Tables

**Table 1:** Series of surfactants added to the organic phase to investigate the stability of W/O/W emulsion droplets.

	**Surfactant added to the organic phase**	**Hydrophile-lipophile balance (HLB) value**
(a)	None	–
(b)	1% (w/w) SPAN 85	1.8
(c)	1% (w/w) SY-Glyster CRS-75	3
(d)	1% (w/w) SPAN 80	4.3
(e)	1% (w/w) SPAN 80 + 0.5% (w/w) SY-Glyster CRS-75	4.3, 3

**Table 2:**
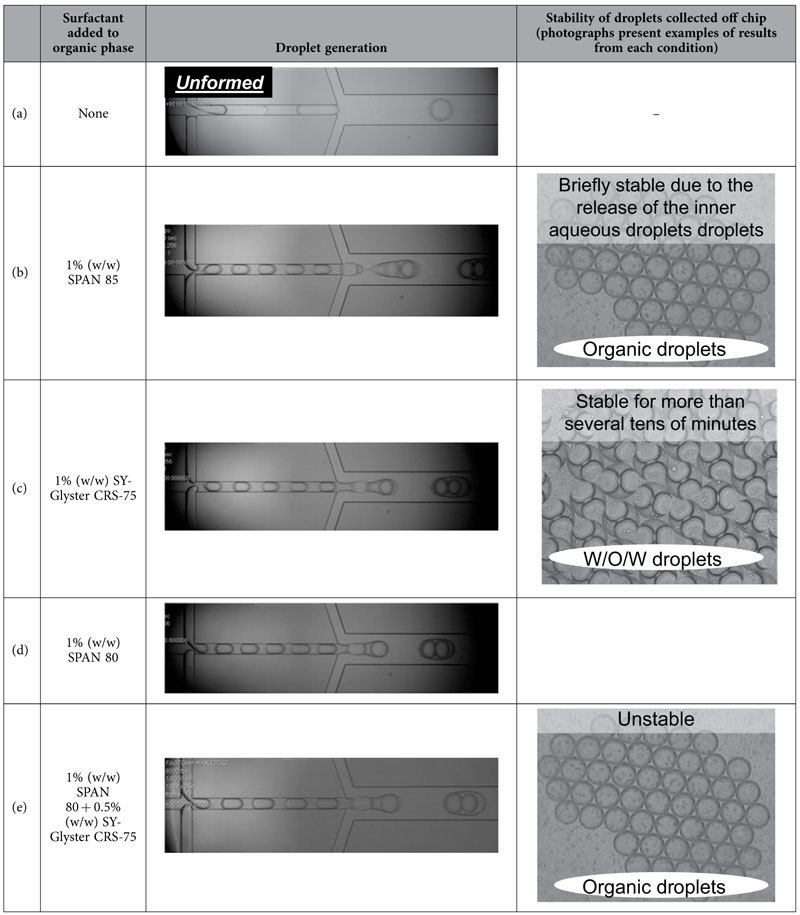
Effect of surfactants on the stability of droplet generation and collected droplets.

**Table 3:** Interfacial tension and spreading coefficient.

**Phase 1**	**Phase 2**	**Phase 3**	**γ12**	**γ13**	**γ23**	**S1**	**S2**	**S3**	**Morphology**
DMEM	Decane/1 % (w/w) SPAN 80	PBS/2 % (w/w) PVA	13.3	4.24	2.53	−15.0	−11.6	6.55	Complete engulfing
DMEM	Decane/1 % (w/w) SPAN 85	PBS/2 % (w/w) PVA	13.3	4.24	2.53	−15.0	−11.6	6.55	Complete engulfing
DMEM	Decane/1 % (w/w) SY-Glyster CRS-75	PBS/2 % (w/w) PVA	13.5	4.24	2.61	−15.1	−11.9	6.65	Complete engulfing
DMEM	Decane/1 % (w/w) SPAN 80 + 0.5 % (w/w) SY-Glyster CRS-75	PBS/2 % (w/w) PVA	13.6	4.24	2.64	−15.2	−12.0	6.69	Complete engulfing
PBS + particle	Decane/1 % (w/w) SY-Glyster CRS-75	PBS/2 % (w/w) PVA	13.3	4.12	2.61	−14.8	−11.8	6.56	Complete engulfing

Interfacial tension was calculated by measurements† of surface tension. ^†^DMEM: 71.6 mN/m, PBS + particle: 71.1 mN/m, Decane/1% (w/w) SPAN 80: 23.1 mN/m, Decane/1% (w/w) SPAN 85: 23.1 mN/m, Decane/1% (w/w) SY-Glyster CRS-75: 22.9 mN/m, Decane/1% (w/w) SPAN 80 + 0.5% (w/w) SY-Glyster CRS-75: 22.8 mN/m, PBS/2% (w/w) PVA: 71.6 mN/m (Temperature: 21 °C).

## References

[b1] Whitesides G. M. The origins and the future of microfluidics. Nature 442, 368–373, doi: 10.1038/Nature05058 (2006). 16871203

[b2] Courtois F. *et al.* Controlling the Retention of Small Molecules in Emulsion Microdroplets for Use in Cell-Based Assays. Anal Chem 81, 3008–3016, doi: 10.1021/ac802658n (2009). 19284775

[b3] Boedicker J. Q., Vincent M. E. & Ismagilov R. F. Microfluidic Confinement of Single Cells of Bacteria in Small Volumes Initiates High-Density Behavior of Quorum Sensing and Growth and Reveals Its Variability. Angew Chem Int Ed 48, 5908–5911, doi: 10.1002/anie.200901550 (2009). PMC2748941 19565587

[b4] Velasco D., Tumarkin E. & Kumacheva E. Microfluidic Encapsulation of Cells in Polymer Microgels. Small 8, 1633–1642, doi: 10.1002/smll.201102464 (2012). 22467645

[b5] Mazutis L. *et al.* Single-cell analysis and sorting using droplet-based microfluidics. Nature protocols 8, 870–891, doi: 10.1038/nprot.2013.046 (2013). 23558786 PMC4128248

[b6] Fei Z. *et al.* Gene Transfection of Mammalian Cells Using Membrane Sandwich Electroporation. Anal Chem 79, 5719–5722, doi: 10.1021/ac070482y (2007). 17600386

[b7] Gel M. *et al.* Dielectrophoretic cell trapping and parallel one-to-one fusion based on field constriction created by a micro-orifice array. Biomicrofluidics 4, doi: 10.1063/1.3422544 (2010). PMC2917888 20697592

[b8] Kemna E. W. M., Wolbers F., Vermes I. & van den Berg A. On chip electrofusion of single human B cells and mouse myeloma cells for efficient hybridoma generation. Electrophoresis 32, 3138–3146, doi: 10.1002/elps.201100227 (2011). 22025094

[b9] Rakszewska A., Tel J., Chokkalingam V. & Huck W. T. S. One drop at a time: toward droplet microfluidics as a versatile tool for single-cell analysis. Npg Asia Mater 6, doi: 10.1038/am.2014.86 (2014).

[b10] Theberge A. B. *et al.* Microfluidic platform for combinatorial synthesis in picolitre droplets. Lab Chip 12, 1320–1326, doi: 10.1039/c2lc21019c (2012). 22344399

[b11] Zheng B., Tice J. D., Roach L. S. & Ismagilov R. F. A droplet-based, composite PDMS/glass capillary microfluidic system for evaluating protein crystallization conditions by microbatch and vapor-diffusion methods with on-chip X-ray diffraction. Angew Chem 43, 2508–2511, doi: 10.1002/anie.200453974 (2004). 15127437 PMC1766324

[b12] Dittrich P. S. & Manz A. Lab-on-a-chip: microfluidics in drug discovery. Nat Rev Drug Discov 5, 210–218 (2006). 16518374 10.1038/nrd1985

[b13] Edd J. F. *et al.* Controlled encapsulation of single-cells into monodisperse picolitre drops. Lab Chip 8, 1262–1264, doi: 10.1039/b805456h (2008). 18651066 PMC2570196

[b14] Cao Z. *et al.* Droplet Sorting Based on the Number of Encapsulated Particles Using a Solenoid Valve. Lab Chip 13, 17–178, doi: 10.1039/c2lc40950j (2012). 23160342

[b15] Okushima S., Nisisako T., Torii T. & Higuchi T. Controlled production of monodisperse double emulsions by two-step droplet breakup in microfluidic devices. Langmuir 20, 9905–9908, doi: 10.1021/la0480336 (2004). 15518471

[b16] Shimamura J., Yokoyama Y., Moriguchi H. & Torii T. in The 14th International Conference on Miniaturized Systems for Chemistry and Life Sciences. 1820–1822, Groningen, The Netherlands (2010).

[b17] Hung L. H. *et al.* Alternating droplet generation and controlled dynamic droplet fusion in microfluidic device for CdS nanoparticle synthesis. Lab Chip 6, 174–178, doi: 10.1039/b513908b (2006). 16450024

[b18] Tamaki S., Wada S., Tsuchiya H., Imran Al-Haq M. & Torii T. in The 7th International Conference on Miniaturized Systems for Chemistry and Life Sciences. 1459–1461, Paris, France (2007).

[b19] Griffin W. C. Classification of surface-active agents by “HLB”. J Soc Cosmet Chem 1, 311–326 (1949).

[b20] Aserin A. Multiple emulsions : technology and applications (Wiley, 2008).

[b21] Torza S. & Mason S. G. Three-phase interactions in shear and electrical fields. J Colloid Interface Sci 33, 67–83, doi: 10.1016/0021-9797(70)90073-1 (1970).

[b22] Xu J.-H., Ge X.-H., Chen R. & Luo G.-S. Microfluidic preparation and structure evolution of double emulsions with two-phase cores. RSC Adv 4, 1900–1906, doi: 10.1039/c3ra46562d (2014).

[b23] Fowkes F. M. ATTRACTIVE FORCES AT INTERFACES. Ind Eng Chem 56, 40–52, doi: 10.1021/ie50660a008 (1964).

